# Tumor tissue-of-origin classification using miRNA-mRNA-lncRNA interaction networks and machine learning methods

**DOI:** 10.3389/fbinf.2025.1571476

**Published:** 2025-05-06

**Authors:** Ankita Lawarde, Masuma Khatun, Prakash Lingasamy, Andres Salumets, Vijayachitra Modhukur

**Affiliations:** ^1^ Department of Obstetrics and Gynecology, Institute of Clinical Medicine, University of Tartu, Tartu, Estonia; ^2^ Celvia CC AS, Tartu, Estonia; ^3^ Department of Obstetrics and Gynecology, University of Helsinki, Helsinki University Central Hospital, Helsinki, Finland; ^4^ Division of Obstetrics and Gynecology, Department of Clinical Science, Intervention and Technology (CLINTEC), Karolinska Institute, and Karolinska University Hospital, Huddinge, Stockholm, Sweden

**Keywords:** miRNAs, network, machine learning, feature selection, tumor tissue origin, ensemble learning

## Abstract

**Introduction:**

MicroRNAs (miRNAs) regulate gene expression and play an important role in carcinogenesis through complex interactions with messenger RNAs (mRNAs) and long non-coding RNAs (lncRNAs). Despite their established influence on tumor progression and therapeutic resistance, the application of miRNA interaction networks for tumor tissue-of-origin (TOO) classification remains underexplored.

**Methods:**

We developed a machine learning (ML) framework that integrates miRNA-mRNA-lncRNA interaction networks to classify tumors by their tissue of origin. Using transcriptomic profiles from 14 cancer types in The Cancer Genome Atlas (TCGA), we constructed co-expression networks and applied multiple feature selection techniques including recursive feature elimination (RFE), random forest (RF), Boruta, and linear discriminant analysis (LDA) to identify a minimal yet informative subset of miRNA features. Ensemble ML algorithms were trained and validated with stratified five-fold cross-validation for robust performance assessment across class distributions.

**Results:**

Our models achieved an overall 99% classification accuracy, distinguishing 14 cancer types with high robustness and generalizability. A minimal set of 150 miRNAs selected via RFE resulted in optimal performance across all classifiers. Furthermore, in silico validation revealed that many of the top miRNAs, including *miR-21-5p, miR-93-5p,* and *miR-10b-5p*, were not only highly central in the network but also correlated with patient survival and drug response. In addition, functional enrichment analyses indicated significant involvement of miRNAs in pathways such as *TGF*-beta signaling, epithelial-mesenchymal transition, and immune modulation. Our comparative analysis demonstrated that models based on miRNA outperformed those using mRNA or lncRNA classifiers.

**Discussion:**

Our integrated framework provides a biologically grounded, interpretable, and highly accurate approach for tumor tissue-of-origin classification. The identified miRNA biomarkers demonstrate strong translational potential, supported by clinical trial overlap, drug sensitivity data, and survival analyses. This work highlights the power of combining miRNA network biology with ML to improve precision oncology diagnostics and supports future development of liquid biopsy-based cancer classification.

## 1 Introduction

Cancer is the second leading cause of death globally, accounting for nearly 9.7 million deaths as of 2022, and is projected to become the leading cause of premature mortality by the end of the century (Bray et al., 2024; Murthy et al., 2024). In the U.S., cancer incidence rates for 2025 are projected at 643.5 per 100,000 males and 581.4 per 100,000 females, with total expected deaths of 618,120, underscoring the increasing cancer burden (Siegel et al., 2025). Despite considerable advancements in research, early detection with accurate classification remains a significant challenge due to non-specific symptoms, eventually leading to late-stage diagnoses and poor survival (Crosby et al., 2022; Pulumati et al., 2023; Ali et al., 2021; Lingasamy et al., 2019; Lingasamy et al., 2021).

MicroRNAs (miRNAs), small non-coding RNAs, typically 17–25 nucleotides long, have gained prominence as cancer biomarkers due to their role as oncogenes or tumor suppressors (Peng and Croce, 2016; Chakrabortty et al., 2023; Pekarek et al., 2023). Identifying cancer-specific miRNA signatures is essential for understanding molecular mechanisms, enabling early-stage detection, and improving treatment outcomes (Peng and Croce, 2016; Yerukala Sathipati et al., 2023; Paranjape et al., 2009; Galvão-Lima et al., 2021). Dysregulated miRNAs significantly impact cellular and biological processes such as apoptosis, cell cycle regulation, metastasis, transcriptome, and interactions within the tumor microenvironment (Peng and Croce, 2016; Sempere et al., 2021; Li et al., 2022). In fact, clinical trials targeting miRNAs (*miR-16, miR-34a, miR-155, and miR-193a-3p*) have shown promising therapeutic potential for various types of cancer (Seyhan, 2024; Kim and Croce, 2023; Qian et al., 2024).

In parallel, long non-coding RNAs (lncRNAs) are recognized as critical regulators of gene expression and tumor progression, thereby expanding the focus of non-coding RNA research in oncology (Huang et al., 2022; Ratti et al., 2020). Certain lncRNAs act as miRNA sponges or competing endogenous RNAs (ceRNAs) that regulate gene expression by competitively binding to shared miRNA-targets through miRNA response elements (MREs), thereby reducing the ability of miRNAs to suppress target mRNA transcripts (Salmena et al., 2011). The advent of high-throughput RNA sequencing facilitates quantitative profiling of miRNA, mRNA, and lncRNA expression levels and aids in identifying MREs in the 3′ untranslated regions (UTRs) of target genes. Tools like TargetScan and miRanda help predict interactions between miRNAs and mRNAs/lncRNAs, revealing regulatory connections that affect cancer pathways (Lewis et al., 2005; Betel et al., 2008). Dysregulation of ceRNAs can lead to abnormal gene expression, promoting cancer progression, metastasis, drug resistance, and maintenance of cancer stem cells (Salmena et al., 2011; Tay et al., 2014; Gutschner and Diederichs, 2012). lncRNAs such as *MALAT1*, a prognostic marker for metastasis in non-small cell lung cancer (NSCLC) (Gutschner and Diederichs, 2012) and *H19* enhance tumorigenesis by influencing tumor suppressor genes and oncogenes (Zhang et al., 2022), while other lncRNAs like *HOTAIR* and *DANCR* facilitate cancer metastasis through ceRNA networks (Cheng and Huang, 2021), underscoring their potential as therapeutic targets and biomarkers for cancer prognosis.

Advances in sequencing technologies, supported by large-scale initiatives like The Cancer Genome Atlas (TCGA) (Tomczak et al., 2015), have potentially enhanced our understanding of cancer genomics. These initiatives have identified miRNA and lncRNA profiles associated with tumor progression, metastasis, and therapy resistance, establishing them as promising biomarkers and therapeutic targets (Dong et al., 2020; Gao and Zhou, 2019; Cho and Han, 2017). For example, *miR-29b/c* in gastric cancer and *miR-186* and *miR-34a* in breast cancer play critical roles in modulating key pathways linked to tumor progression (Gong et al., 2014; Sun et al., 2019). Similarly, lncRNAs like *HOTAIR* and *MALAT1* have been implicated in promoting invasion and metastasis (Archit et al., 2024; Zhou et al., 2021). In prostate cancer, the negative correlation between *miR-142-5p* and lncRNA *ADAMTS9-AS1* has been found to facilitate tumor progression (Archit et al., 2024; Zhou et al., 2021).

Integrating miRNA expression data into pan-cancer analyses can identify shared molecular signatures across diverse cancer types (Mitra et al., 2020; Lopez-Rincon et al., 2020; Tan et al., 2019). Pan-cancer studies increasingly use miRNAs as biomarkers for cancer classification and TOO prediction, achieving 84.27% accuracy for cancer type classification (Yerukala Sathipati et al., 2023) and 97% accuracy for predicting TOO in metastatic cancers using neural networks (Raghu et al., 2024). Furthermore, treatment strategies guided by miRNAs based on support vector machines (SVM) showed a higher classification accuracy of 97.2% (Cheerla and Gevaert, 2017). However, the above methods often overlook the intricate interactions of miRNAs within broader molecular networks, which is crucial for capturing the complexity of cancer biology. Thus, multi-omics models integrating miRNA, mRNA, and lncRNA data may offer promising potential for pan-cancer classification.

To address the current limitations, our study utilized comprehensive machine learning (ML) methods to classify TOO using miRNAs identified from miRNA-mRNA-lncRNA interactions from TCGA datasets. Further, we identified a minimal set of 150 miRNA biomarkers using ensemble ML methods, achieving 99% accuracy in distinguishing 14 cancer types. Our findings are projected to emphasize the potential of integrating computational and biological approaches to advance precision oncology, enabling the development of innovative diagnostic tools and treatment strategies.

## 2 Materials and methods

### 2.1 Data collection and preprocessing

Transcriptomic profiles, including miRNA-Seq (miRNA isoform) and RNA-Seq data, were obtained from the TCGA project via the Genomics Data Commons portal (GDC) (Heath et al., 2021). Raw read counts for both solid tissue normal (NT) and primary solid tumor (TP) tissue samples were downloaded using the Bioconductor R package TCGAbiolinks (v2.32.0). The selection of cancer types was limited to those with at least 10 patient samples per cancer type, with data for both tumor and corresponding normal tissues included. To maintain consistency, only primary tumor samples were analyzed. The disease type classifications, along with their respective primary sites and TCGA project names, are summarized in Table 1. A comprehensive breakdown of sample counts (NT and TP samples per cancer) for miRNA-Seq and RNA-Seq datasets, categorized by cancer type and tissue type, is provided in Table 1. In total, the dataset comprised 14 cancer types, with 6,485 and 6,507 samples from miRNA-Seq and RNA-Seq distributed across tumor tissues and 640 and 660 samples from miRNA-Seq and RNA-Seq distributed across normal tissues, respectively.

**TABLE 1:** Projects and cancer types from TCGA.

Project	Project name	Disease type	Primary site	NT miRNA-Seq	TP miRNA-Seq	Total count miRNA-Seq	NT RNA-Seq	TP RNA-Seq	Total count RNA-Seq
TCGA-BLCA	Bladder Urothelial Carcinoma	Adenomas and Adenocarcinomas	Bladder	19	417	436	19	412	431
		Epithelial Neoplasms, NOS							
		Squamous Cell Neoplasms							
		Transitional Cell Papillomas and Carcinomas							
TCGA-BRCA	Breast Invasive Carcinoma	Adenomas and Adenocarcinomas	Breast	104	1094	1198	113	1111	1224
		Adnexal and Skin Appendage Neoplasms							
		Basal Cell Neoplasms							
		Complex Epithelial Neoplasms							
		Cystic, Mucinous and Serous Neoplasms							
		Ductal and Lobular Neoplasms							
		Epithelial Neoplasms, NOS							
		Fibroepithelial Neoplasms							
		Squamous Cell Neoplasms							
TCGA-ESCA	Esophageal Carcinoma	squamous cell neoplasms	Esophagus	13	186	199	13	184	197
		adenomas and adenocarcinomas							
		cystic, mucinous and serous neoplasms							
		adenomas and adenocarcinomas	Stomach						
		squamous cell neoplasms							
TCGA-HNSC	Head and Neck Squamous Cell Carcinoma	squamous cell neoplasms	Base of tongue	44	523	567	44	520	564
			Bones, joints and articular cartilage of other and unspecified sites						
			Floor of mouth						
			Gum						
			Hypopharynx						
			Larynx						
			Lip						
			Oropharynx						
			Other and ill-defined sites in lip, oral cavity and pharynx						
			Other and unspecified parts of mouth						
			Other and unspecified parts of tongue						
			Palate						
			Tonsil						
TCGA-KICH	Kidney Chromophobe	Adenomas and Adenocarcinomas	Kidney	25	66	91	25	66	91
TCGA-KIRP	Kidney Renal Papillary Cell Carcinoma		Kidney	34	291	325	32	290	322
TCGA-KIRC	Kidney Renal Clear Cell Carcinoma		Kidney	71	544	615	72	541	613
TCGA-LIHC	Liver Hepatocellular Carcinoma	Adenomas and Adenocarcinomas	Liver and intrahepatic bile ducts	50	372	422	50	371	421
TCGA-LUAD	Lung Adenocarcinoma	Acinar Cell Neoplasms	Bronchus and lung	46	519	565	59	539	598
		Adenomas and Adenocarcinomas							
		Cystic, Mucinous and Serous Neoplasms							
TCGA-LUSC	Lung Squamous Cell Carcinoma	Squamous Cell Neoplasms	Bronchus and lung	45	478	523	51	502	553
TCGA-STAD	Stomach Adenocarcinoma	Adenomas and Adenocarcinomas	Stomach	45	446	491	36	412	448
		Cystic, Mucinous and Serous Neoplasms							
TCGA-PRAD	Prostate Adenocarcinoma	Adenomas and Adenocarcinomas	Prostate gland	52	498	550	52	501	553
		Cystic, Mucinous and Serous Neoplasms							
		Ductal and Lobular Neoplasms							
TCGA-THCA	Thyroid Carcinoma	Adenomas and Adenocarcinomas	Thyroid gland	59	506	565	59	505	564
		Epithelial Neoplasms, NOS							
TCGA-UCEC	Uterine Corpus Endometrial Carcinoma	Adenomas and Adenocarcinomas	Corpus uteri	33	545	578	35	553	588
		cystic, mucinous and serous neoplasms							
		epithelial neoplasms, nos							
		not reported	Uterus, NOS	640	6485	7125	660	6507	7167
			Total						

Projects and cancer types from TCGA, along with the sample count of miRNA/RNA, expression profiles per tissue and cancer types used in this study. (NT: Solid tissue normal TP: Primary tumor tissue).

### 2.2 miRNA network construction

The construction of miRNA networks for each cancer was based on the methodology outlined earlier (Lawarde et al., 2024). The procedure can be summarized as follows:

#### 2.2.1 Differential expression analysis

We conducted differential expression analysis between tumor and normal tissue using the R package DESeq2 (v1.44.0), for miRNA-Seq data and applied VST to visualize the data using t-SNE plot. Expression matrices for protein-coding genes and lncRNAs for each type of cancer were also extracted from the RNA-Seq dataset to perform differential expression analysis using the same DESeq2 package.

#### 2.2.2 Network construction

After identifying common patient samples shared between both miRNA-Seq and RNA-Seq datasets, we calculated Pearson correlation coefficients to construct a miRNA-mRNA-lncRNA co-expression correlation network. This network included miRNAs, mRNA, and lncRNAs that met the criteria |log2 fold change| ≥ 1, p-values adjusted using the Benjamini–Hochberg (BH) method was <0.05, and the correlation coefficient |R| ≥ 0.5. The R package igraph (v2.1.1) was utilized for network construction, and the fast greedy algorithm identified communities within the network. Additionally, the assortativity coefficient and the degree of the network were calculated using the igraph package. The miRNA features were obtained from the edge table of each cancer type, as shown in (Supplementary Table S1).

### 2.3 Machine learning models to classify multiple cancer types based on TOO

A classification model using interacting miRNAs was developed to categorize 27 different classes, including 7,125 samples (training samples 4,978), consisting of NT and TP samples and cancer types as detailed in Table 1, using tree-based and ensemble machine learning techniques. A minimum sample requirement of 15 was established to ensure robust model training and testing, focusing exclusively on these samples. Due to insufficient data, NT samples from the esophageal carcinoma (ESCA) were excluded from further analysis.

#### 2.3.1 Machine learning (ML) methods used for training

We implemented four different machine-learning methods to train our classification models as described below:

1.Random Forest (RF): RF builds multiple decision trees using bootstrapped samples and selected features randomly, improving accuracy and enhancing robustness while avoiding overfitting. Moreover, the RF method aggregates the results from individual trees to provide a more stable and accurate prediction.
2.AdaBoost (Adaptive Boosting): AdaBoost sequentially combines weak classifiers, focusing on previously misclassified instances. However, AdaBoost is sensitive to noise and outliers despite effective reduction of bias, impacting the overall performance in certain datasets.
3.XGBoost (Extreme Gradient Boosting): XGBoost refines gradient boosting through efficient parallel processing and regularization, making it particularly suitable for high-dimensional datasets.
4.LightGBM (Light Gradient Boosting Machine): LightGBM is yet another gradient boosting approach that increases speed and memory efficiency by using histogram-based learning and leaf-wise growth techniques, making it particularly effective for larger datasets. Together, these ensemble methods used in our models leverage the strengths of multiple models, thereby enhancing predictive performance and robustness across various machine-learning tasks.

#### 2.3.2 Training and test set

The miRNA expression dataset, encompassing 14 cancer types and 27 classes, was divided into training and test sets with a 70:30 split. Specifically, 70% of the data was allocated for model training, while the remaining 30% was reserved for testing. The model was trained and tested using Python 3. A pipeline was constructed using the imblearn.pipeline module, which included StandardScaler from sklearn.preprocessing for feature scaling and the Synthetic Minority Over-sampling Technique (SMOTE) technique from imblearn.over_sampling to address class imbalance. This pipeline was used for training, integrating feature scaling and sample balancing. Further, all the prediction models were cross-validated using a 5-fold strategy with the StratifiedKFold method from the sklearn.model_selection module. The classification report and confusion matrix were generated using the classification_report and confusion_matrix functions from the sklearn.metric module. The Area Under the Curve (AUC) was calculated using the roc_auc_score function from the same module.

All methods were implemented with default parameters, except for the AdaBoost method, which was tuned through hyperparameter adjustment. Specifically, we used a decision tree as the base estimator with a maximum depth of 5, set the algorithm to Stagewise Additive Modeling using a Multiclass Exponential loss function (SAMME), adjusted the learning rate to 1.2, and set the number of estimators to 300 for the AdaBoost model.

### 2.4 Feature selection

We employed four feature selection methods on all interacting miRNAs to identify the most relevant predictors for the classification task. Recursive Feature Elimination (RFE) was used to iteratively remove the least important features based on model performance, effectively narrowing down the feature set. The Boruta method, a wrapper algorithm, was applied to determine the significance of features by comparing their importance to random permutations, ensuring only the most relevant features. Linear Discriminant Analysis (LDA) was also utilized to select features that maximally separate between classes, focusing on those contributing to the best class discrimination. Finally, the Random Forest (RF) method provided feature importance scores, allowing for the selection of features based on their contribution to the predictive power of the model accuracy. This comprehensive approach of feature selection improved model performance and minimized dimensionality, ensuring that only the most relevant features were utilized for multiclass classification.

### 2.5 Model evaluation metrics

To evaluate the performance of each classification model, we used standard metrics, including accuracy, sensitivity, specificity, precision, F1-score, and AUC, in line with similar studies (Modhukur et al., 2021; Rahmani et al., 2023). The performance metrics were computed as follows:

•**Precision** = *TP/(TP + FP)*
•**Recall/Sensitivity** = *TP/(TP + FN)*
•**F1-score** = 2*TP/(2 * TP + FP + FN)
•**Accuracy** = *(TP + TN)/(TP + TN + FP + FN)*
•**Sensitivity** = *TP/(TP + FN)*
•**AUC:**AUC refers to the area under the Receiver Operating Characteristic (ROC) curve.

AUC provides an aggregate measure of performance across all classification thresholds, indicating the model’s ability to distinguish between classes effectively.

### 2.6 Cross-validation of interacting miRNAs with literature and clinical trial data

We manually compiled a comprehensive collection of miRNAs in cancer, miRNA isoforms in cancer, extracellular vesicular (EV) miRNAs, and clinical trial miRNAs from the literature (Supplementary File S1). Additionally, we downloaded the miRNA-drug associations from the noncoRNA db (Li et al., 2020) and miRNA genes from the Cancer miRNA Census (CMC miRNAs) from the published paper (Suszynska et al., 2024). The CMC miRNA genes were mapped to miRNA IDs (miRBase v21) and overlapping miRNAs between CMC and all interacting miRNAs were identified. Our literature-derived compendium and drug-target association were visualized with Venn diagrams and pie charts using R packages ggplot2 (v3.5.1) and VennDiagram (v1.7.3).

### 2.7 Machine learning classifier comparison with other biomolecules: mRNAs and lncRNAs

The LightGBM model was trained on three sets of mRNA features. The mRNA features were selected from the interactions between the miRNA-mRNA-lncRNA network. All mRNAs are significantly regulated in each cancer type (|log2FoldChange| ≥ 1 and adjust p-value with BH < 0.05) (all interactions are listed in Supplementary Table S1). We used random number generation to pick the number of mRNA features from a total of 6207 interacting mRNAs. Two random numbers, 123 and 223, were selected from 100 to 200 and 200 to 300 random numbers. Similarly, for lncRNAs, we used random number generation to select lncRNA features from a total of 2245 lncRNAs to train the ML models. A total of 105 and 258 lncRNAs were selected from 100 to 200 and 200 to 300 random numbers. The training steps are followed in the same manner as mentioned for the miRNA models above. For both mRNAs and lncRNAs, we trained three models each. Two from random feature selection and one with all interacting mRNAs/lncRNAs.

### 2.8 Functional enrichment analysis

We obtained experimentally validated gene targets of interacting miRNAs from TarBase, miRTarBase, and miRecords databases using the Bioconductor R package multiMiR (v1.26.0). Gene Ontology (GO) and Kyoto Encyclopedia of Genes and Genomes (KEGG) enrichment analyses were conducted for these gene targets using the Bioconductor package clusterProfiler (v4.12.6), and the results were visualized as a dot plot. Additionally, GO and KEGG enrichment analyses were performed for protein-coding and lncRNA genes obtained from each cancer type’s network. Finally, we conducted a separate enrichment analysis for the targets of common miRNAs compiled from all interacting miRNAs, miRNA compendium (including EVmiRNA list), and CMC (Suszynska et al., 2024) miRNAs.

### 2.9 Survival and clinical prognosis analysis of interacting miRNAs

To evaluate the prognostic potential of the identified interacting miRNAs, we performed univariate Cox-PH analysis using the MethSurv pipeline (Modhukur et al., 2018). Patients were stratified into high- and low-expression groups based on the median expression level of each miRNA. The statistical significance of the association between miRNA expression and overall survival was assessed using the log-rank test. The proportional hazards assumption was verified using the Schoenfeld residuals test, and survival curves were visualized with the Kaplan-Meier (KM) plot. We used the R packages survival (v3.7.0) and survminer (v0.4.9) for survival analysis and visualizations, respectively.

## 3 Results

### 3.1 Workflow overview

**FIGURE 1:**
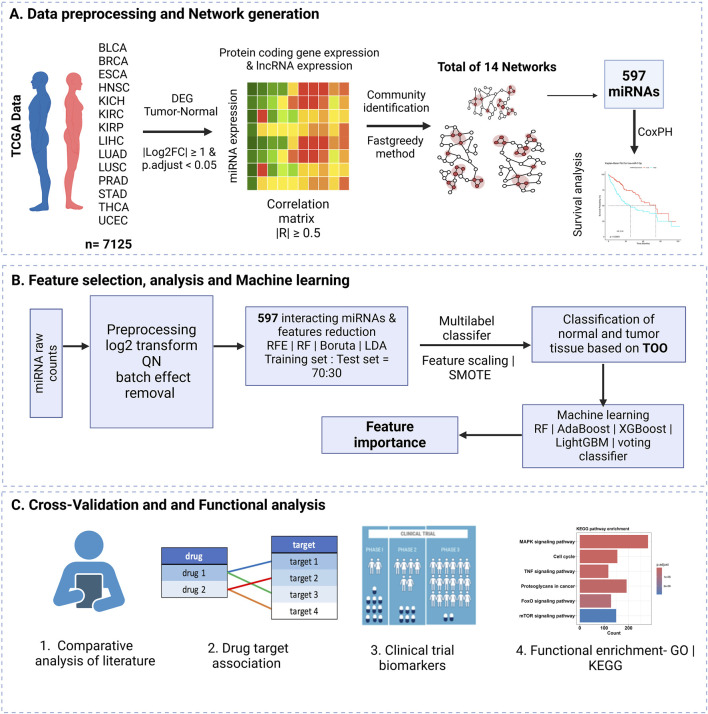
Overview of the study workflow. **(A)** Data preprocessing and network creation from TCGA data for 14 cancers were included in the study, resulting in 14 networks and 597 miRNAs selected from the highly correlated networks. **(B)** Feature selection and model training using network-derived features. **(C)** Cross-validation of selected features through literature and database comparisons.

The overview of the workflow adopted in this study is illustrated in Figure 1, which consists of three main sections, as summarized below.

(A) Data Collection and Preprocessing
  i.Raw read counts were collected from TCGA for 14 cancer types, with a focus on miRNAs, protein-coding genes, and lncRNAs.
  ii.Differential expression analysis was performed using DESeq2, comparing tumor and normal samples with strict significance thresholds (|log2 fold change| ≥ 1 and adjusted p-value <0.05).
  iii.The raw counts were normalized using variance stabilizing transformation (VST) to reduce heteroscedasticity and improve comparability across samples. The VST-normalized data was used for downstream visualizations, including t-SNE plots.
  iv.A Pearson correlation matrix was created to evaluate relationships between differentially regulated miRNAs, mRNAs, and lncRNAs to help identify potential interactions.
  v.miRNA-mRNA-lncRNA network was constructed based on the aforementioned correlations, considering only interactions with a correlation coefficient (|R|) of 0.5 or higher.
  vi.The network structure was analyzed further through community identification using the fast-greedy method, revealing clusters of interacting features.
  vii.A total of 597 interacting miRNAs were selected for subsequent analysis.
  viii.Survival analysis was performed using univariate Cox Proportional Hazards (Cox-PH) regression to assess the relationship between miRNA expression and patient survival.
(B) Feature Selection, Analysis, and Machine Learning
  i.The raw miRNA counts were log2 transformed, quantile normalized, and batch effects removed. From the total preprocessed data, a subset of the quantile normalized count matrix of 597 interacting miRNA obtained from part (A) was used for the next steps.
  ii.Dimensionality was reduced by using feature selection methods: RFE, RF, Boruta, and LDA.
  iii.The data were split into 70% training and 30% testing sets, followed by feature scaling and application of SMOTE to address class imbalance.
  iv.A multilabel classification model was used to classify normal and tumor tissues, employing machine learning algorithms including RF, Adaptive Boosting (AdaBoost), Extreme Gradient Boosting (XGBoost), Light Gradient Boosting Machine (LightGBM), and a voting classifier, along with feature importance evaluations.
(C) Validation with Literature and Functional Enrichment Analysis
  i.The results were validated through comparisons with existing literature.

Functional enrichment analysis, including Gene Ontology (GO) and Kyoto Encyclopedia of Genes and Genomes (KEGG) pathway enrichment, were used to identify miRNA-drug target associations and potential biomarkers in clinical trials.

### 3.2 Overview of interacting miRNAs and network properties

In our analysis, a highly correlated network (|R| ≥ 0.5) of differentially regulated miRNA, mRNA, and lncRNA for the selected cancer types, applying significant thresholds of p. adjust <0.05 and |log2FoldChange| ≥1) was constructed, where the assortativity coefficient and degree of assortativity for networks ranged from −0.59 to −0.85 and −0.3 to −0.63, respectively. The negative assortativity coefficient indicates that the nodes tend to connect to other nodes with different properties, such as high-degree nodes (miRNAs) connecting with low-degree nodes (protein-coding genes and lncRNAs). After removing duplicates, a total of 597 unique miRNAs were compiled by combining miRNAs from networks of all 14 cancer types. The top ten miRNAs with the highest degree of centrality per cancer type are presented in Table 2, with a detailed list of interactions per cancer shown in Supplementary Table S1. Key miRNAs, including *miR-145-3p/5p, miR-142-3p, miR-100-5p, miR-143-3p,* and *miR-199b-5p*, exhibited the highest degree of centrality across multiple cancer types in our study, highlighting their potential involvement in oncogenic pathways.

**TABLE 2:** Degree centrality of miRNAs in selected cancer types.

BLCA (bladder urothelial carcinoma)	Degree centrality	BRCA (breast invasive carcinoma)	Degree centrality
hsa-miR-125b-5p	362	hsa-miR-190b	197
hsa-miR-145-3p	353	hsa-miR-155-5p	137
hsa-let-7c-5p	323	hsa-miR-934	135
hsa-miR-99a-5p	297	hsa-miR-18a-5p	118
hsa-miR-100-5p	295	hsa-miR-142-5p	106
hsa-miR-143-3p	282	hsa-miR-577	88
hsa-miR-145-5p	280	hsa-miR-379-5p	54
hsa-miR-6507-5p	242	hsa-miR-135b-5p	45
hsa-miR-200a-3p	225	hsa-miR-199b-5p	43
hsa-miR-141-3p	220	hsa-miR-142-3p	41

LIHC (Liver Hepatocellular Carcinoma)	Degree centrality	LUAD (Lung Adenocarcinoma)	Degree centrality
hsa-miR-105-5p	226	hsa-miR-34b-3p	263
hsa-miR-767-5p	224	hsa-miR-34c-3p	248
hsa-miR-4652-5p	189	hsa-miR-34b-5p	229
hsa-miR-4746-5p	174	hsa-miR-105-5p	108
hsa-miR-767-3p	142	hsa-miR-767-5p	108
hsa-miR-199a-3p	132	hsa-miR-150-5p	89
hsa-miR-199b-3p	132	hsa-miR-767-3p	80
hsa-miR-466	110	hsa-miR-548y	69
hsa-miR-199b-5p	109	hsa-miR-194-5p	65
hsa-miR-214-3p	86	hsa-miR-192-5p	61

ESCA (Esophageal Carcinoma)	Degree centrality	HNSC (Head and Neck Squamous Cell Carcinoma)	Degree centrality
hsa-miR-944	930	hsa-miR-133a-5p	297
hsa-miR-205-3p	833	hsa-miR-1-5p	277
hsa-miR-205-5p	813	hsa-miR-1-3p	264
hsa-miR-149-5p	731	hsa-miR-133b	261
hsa-miR-6499-3p	609	hsa-miR-133a-3p	257
hsa-miR-708-5p	588	hsa-miR-499a-5p	245
hsa-miR-708-3p	582	hsa-miR-206	229
hsa-miR-375	553	hsa-miR-381-3p	103
hsa-miR-224-5p	495	hsa-miR-9-5p	99
hsa-miR-452-5p	484	hsa-miR-193b-3p	77
hsa-miR-142-3p	146	hsa-miR-222-3p	632
hsa-miR-944	70	hsa-miR-221-3p	630
hsa-miR-203a-3p	41	hsa-miR-23b-3p	430
hsa-miR-100-5p	37	hsa-miR-27b-3p	421
hsa-miR-205-5p	33	hsa-miR-145-3p	377
hsa-miR-29c-3p	22	hsa-miR-133b	357
hsa-miR-7702	21	hsa-miR-96-5p	241
hsa-miR-148a-3p	19	hsa-miR-141-3p	223
hsa-miR-196a-5p	13	hsa-miR-143-3p	201
hsa-miR-511-5p	12	hsa-miR-6510-3p	196

KICH (Kidney Chromophobe)	Degree centrality	KIRC (Kidney Renal Clear Cell Carcinoma)	Degree centrality
hsa-miR-221-3p	594	hsa-miR-142-5p	277
hsa-miR-182-5p	474	hsa-miR-155-5p	247
hsa-miR-96-5p	432	hsa-miR-142-3p	133
hsa-miR-222-3p	398	hsa-miR-892b	128
hsa-miR-221-5p	365	hsa-miR-892c-3p	117
hsa-miR-30e-5p	332	hsa-miR-888-5p	111
hsa-miR-455-3p	327	hsa-miR-204-5p	102
hsa-miR-891a-5p	304	hsa-miR-891b	93
hsa-miR-222-5p	293	hsa-miR-892a	87
hsa-miR-29a-3p	240	hsa-miR-21-5p	67

STAD (Stomach Adenocarcinoma)	Degree centrality	THCA (Thyroid Carcinoma)	Degree centrality
hsa-miR-195-5p	605	hsa-miR-146b-3p	892
hsa-miR-100-5p	593	hsa-miR-21-5p	853
hsa-miR-145-3p	570	hsa-miR-146b-5p	853
hsa-miR-125b-5p	569	hsa-miR-7-2-3p	696
hsa-miR-145-5p	548	hsa-miR-1179	672
hsa-miR-133a-3p	528	hsa-miR-204-5p	562
hsa-let-7c-5p	500	hsa-miR-7156-5p	402
hsa-miR-218-5p	487	hsa-miR-375	401
hsa-miR-1-3p	480	hsa-miR-31-3p	374
hsa-miR-133b	448	hsa-miR-6860	366
hsa-miR-143-3p	263	hsa-miR-145-3p	185
hsa-miR-126-3p	262	hsa-miR-145-5p	172
hsa-miR-143-5p	225	hsa-miR-142-5p	94
hsa-miR-145-5p	191	hsa-miR-449a	93
hsa-miR-145-3p	188	hsa-miR-449c-5p	84
hsa-miR-223-3p	179	hsa-miR-449b-5p	81
hsa-miR-199a-3p	141	hsa-miR-449b-3p	81
hsa-miR-199b-3p	138	hsa-miR-199a-5p	80
hsa-miR-1-3p	122	hsa-miR-199b-5p	78
hsa-miR-4772-3p	122	hsa-miR-142-3p	78

Top 10 miRNAs, with the highest degree of centrality for each cancer type in the study.

### 3.3 Comparison of interacting and feature-selected miRNAs

Feature selection methods identified the most representative miRNA signature for cancer-type classification when applied to 597 interacting miRNAs. The RF method identified 298 miRNAs of the most important features based on a median importance (≥0.00039) cutoff, whereas RFE identified 150 miRNAs. The Boruta method yielded 530 miRNA features, while LDA selected 352 miRNAs, achieving 90% cumulative importance. The miRNAs from each feature set are listed in Supplementary Table S2, while Table 3 shows the count of miRNAs in each feature set. The Venn diagram (Supplementary Figure S1) shows the overlap among the 597 interacting miRNAs and those identified by the feature selection methods (RF, LDA, RFE, and Boruta). A total of 98 miRNAs were common between the 597 interacting miRNAs and miRNA feature sets identified by four feature selection methods. Additionally, 40 miRNAs were unique to the 597 miRNA feature set, while 52 miRNAs were shared among the RFE, Boruta, RF, and 597 feature sets.

**TABLE 3:** Total number of miRNAs in each feature set.

Feature set	Interacting miRNAs	RFE	RF	Boruta	LDA
No. Of miRNAs	597	150	298	530	352

### 3.4 Performance of machine learning models

To assess predictive performance, we trained a total of 25 ML models using five algorithms (RF, AdaBoost, XGBoost, LightGBM, and a voting classifier) across five feature sets (597 miRNAs, RFE-selected features, RF-selected features, Boruta-selected features, and LDA-selected features). The model performance was evaluated using precision, recall, and F1-score, with detailed classification results shown in Table 4.

**TABLE 4:** Machine learning model performances with each feature set.

ML models	Score	597			RFE			RF			Boruta			LDA		
		Precision	Recall	f1-score	Precision	Recall	f1-score	Precision	Recall	f1-score	Precision	Recall	f1-score	Precision	Recall	f1-score
Random forest	accuracy	0.99	0.99	0.99	0.99	0.99	0.99	0.99	0.99	0.99	0.99	0.99	0.99	0.99	0.99	0.99
	macro avg	0.98	0.95	0.96	0.98	0.96	0.97	0.99	0.97	0.98	0.99	0.96	0.97	0.99	0.96	0.97
	weighted avg	0.99	0.99	0.99	0.99	0.99	0.99	0.99	0.99	0.99	0.99	0.99	0.99	0.99	0.99	0.99
AdaBoost	accuracy	0.99	0.99	0.99	0.99	0.99	0.99	0.99	0.99	0.99	0.99	0.99	0.99	0.99	0.99	0.99
	macro avg	0.99	0.94	0.96	0.99	0.95	0.96	0.99	0.95	0.97	0.99	0.96	0.97	0.99	0.93	0.96
	weighted avg	0.99	0.99	0.99	0.99	0.99	0.99	0.99	0.99	0.99	0.99	0.99	0.99	0.99	0.99	0.99
XGBoost	accuracy	0.99	0.99	0.99	0.99	0.99	0.99	0.99	0.99	0.99	0.99	0.99	0.99	0.99	0.99	0.99
	macro avg	0.98	0.95	0.96	0.97	0.95	0.96	0.98	0.96	0.97	0.97	0.95	0.96	0.98	0.95	0.96
	weighted avg	0.99	0.99	0.99	0.99	0.99	0.99	0.99	0.99	0.99	0.99	0.99	0.99	0.99	0.99	0.99
LightGBM	accuracy	0.99	0.99	0.99	0.99	0.99	0.99	0.99	0.99	0.99	0.99	0.99	0.99	0.99	0.99	0.99
	macro avg	0.99	0.95	0.97	0.98	0.96	0.97	0.98	0.96	0.97	0.98	0.95	0.96	0.99	0.95	0.97
	weighted avg	0.99	0.99	0.99	0.99	0.99	0.99	0.99	0.99	0.99	0.99	0.99	0.99	0.99	0.99	0.99
Ensemble classifier	accuracy	0.99	0.99	0.99	0.99	0.99	0.99	0.99	0.99	0.99	0.99	0.99	0.99	0.99	0.99	0.99
	macro avg	0.99	0.95	0.97	0.98	0.97	0.97	0.98	0.96	0.97	0.98	0.96	0.97	0.99	0.96	0.97
	weighted avg	0.99	0.99	0.99	0.99	0.99	0.99	0.99	0.99	0.99	0.99	0.99	0.99	0.99	0.99	0.99

Precision, recall, f-score of classification accuracy for 5 machine learning methods, Random Forest, AdaBoost, XGBoost, LightGBM, and ensemble classifier used in the study. (RFE: recursive feature elimination, RF: random forest, LDA: Linear discriminant analysis).

The RF model exhibited consistently high accuracy across all feature sets, achieving an average accuracy of 99.18% ± 0.0013. However, its performance was lowest for the bladder urothelial carcinoma (BLCA) solid tissue normal (NT) (BLCA-NT) class, where recall ranged between 50% and 60%, and F1 scores ranged from 60% to 80%, regardless of the feature set (Supplementary Table S3).

Similarly, the AdaBoost model achieved an overall accuracy of 98.93% ± 0.0017, with the Boruta feature set (530 miRNAs) outperforming others by yielding the highest accuracy of 99.16% (Supplementary Table S4). The XGBoost model achieved an accuracy of 98.80% ± 0.0012, with the RF-selected feature set (298 miRNAs) providing the best performance with 99.02% accuracy (Supplementary Table S5). For LightGBM models, the overall accuracy was 98.98% ± 0.0006. The RF, RFE, and LDA feature sets (298, 150, and 352 miRNAs, respectively) outperformed the 597 (accuracy = 99%) and Boruta feature sets (with 530 miRNAs, the accuracy = 99%). However, the LightGBM models exhibited lower recall for the BLCA-NT and the lung adenocarcinoma (LUAD-NT) classes (Supplementary Table S6).

**FIGURE 2:**
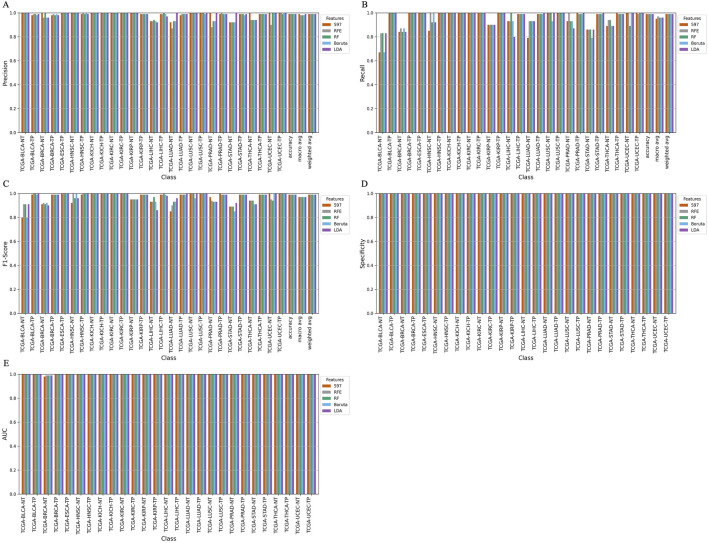
Barplot depicting ensemble model performance across five feature sets. **(A)** Precision and accuracy for each class. **(B)** Recall for each class. **(C)** F1-score for each class. **(D)** Specificity for each class. **(E)** Area under the curve (AUC) for each class.

Furthermore, we developed a voting classifier that combined RF, AdaBoost, XGBoost, and LightGBM models and achieved an average accuracy of 99.03% ± 0.0005. Notably, the RFE feature set (150 miRNAs) demonstrated particularly strong results, achieving 99% accuracy with both weighted and macro averages at 99%. A detailed comparison of model performance metrics as shown in Tables 5–9, and accuracy, precision, recall, F1-score, specificity, and AUC for each class and five feature sets are presented in Figure 2.

**TABLE 5:** Performance of Ensemble classifier with 597 miRNA features.

Response class	597_precision	597_recall	597_f1-score	597_Specificity	597_AUC
TCGA-BLCA-NT	1	0.67	0.8	1	1
TCGA-BLCA-TP	0.98	1	0.99	1	1
TCGA-BRCA-NT	1	0.84	0.91	1	0.98
TCGA-BRCA-TP	0.98	1	0.99	1	1
TCGA-ESCA-TP	1	1	1	1	1
TCGA-HNSC-NT	1	0.85	0.92	1	1
TCGA-HNSC-TP	0.99	1	0.99	1	1
TCGA-KICH-NT	1	1	1	1	1
TCGA-KICH-TP	1	1	1	1	1
TCGA-KIRC-NT	1	1	1	1	1
TCGA-KIRC-TP	1	1	1	1	1
TCGA-KIRP-NT	1	0.9	0.95	1	1
TCGA-KIRP-TP	0.99	1	0.99	1	1
TCGA-LIHC-NT	0.93	0.93	0.93	1	1
TCGA-LIHC-TP	0.99	0.99	0.99	1	1
TCGA-LUAD-NT	0.92	0.79	0.85	1	1
TCGA-LUAD-TP	0.98	0.99	0.99	1	1
TCGA-LUSC-NT	1	1	1	1	1
TCGA-LUSC-TP	1	1	1	1	1
TCGA-PRAD-NT	1	0.93	0.97	1	1
TCGA-PRAD-TP	0.99	1	1	1	1
TCGA-STAD-NT	0.92	0.86	0.89	1	1
TCGA-STAD-TP	0.99	0.99	0.99	1	1
TCGA-THCA-NT	1	0.89	0.94	1	1
TCGA-THCA-TP	0.99	1	0.99	1	1
TCGA-UCEC-NT	1	1	1	1	1
TCGA-UCEC-TP	1	1	1	1	1
Accuracy	0.99	0.99	0.99		
macro avg	0.99	0.95	0.97		
weighted avg	0.99	0.99	0.99		

Precision, recall, f1-score, specify, and AUC, for ensemble classifier using 597 interacting miRNAs, as a feature set.

**TABLE 6:** Performance of Ensemble classifier with RFE miRNA features.

Response class	RFE_precision	RFE_recall	RFE_f1-score	RFE_Specificity	RFE_AUC
TCGA-BLCA-NT	1	0.83	0.91	1	1
TCGA-BLCA-TP	0.99	1	1	1	1
TCGA-BRCA-NT	0.96	0.87	0.92	1	0.99
TCGA-BRCA-TP	0.99	1	0.99	1	1
TCGA-ESCA-TP	1	1	1	1	1
TCGA-HNSC-NT	1	1	1	1	1
TCGA-HNSC-TP	1	1	1	1	1
TCGA-KICH-NT	1	1	1	1	1
TCGA-KICH-TP	1	1	1	1	1
TCGA-KIRC-NT	1	1	1	1	1
TCGA-KIRC-TP	1	1	1	1	1
TCGA-KIRP-NT	1	0.9	0.95	1	1
TCGA-KIRP-TP	0.99	1	0.99	1	1
TCGA-LIHC-NT	0.93	0.93	0.93	1	1
TCGA-LIHC-TP	0.99	0.99	0.99	1	1
TCGA-LUAD-NT	0.87	0.93	0.9	1	1
TCGA-LUAD-TP	0.99	0.99	0.99	1	1
TCGA-LUSC-NT	1	1	1	1	1
TCGA-LUSC-TP	1	1	1	1	1
TCGA-PRAD-NT	0.88	1	0.94	1	1
TCGA-PRAD-TP	1	0.99	0.99	1	1
TCGA-STAD-NT	0.92	0.86	0.89	1	1
TCGA-STAD-TP	0.99	0.99	0.99	1	1
TCGA-THCA-NT	0.94	0.94	0.94	1	1
TCGA-THCA-TP	0.99	0.99	0.99	1	1
TCGA-UCEC-NT	0.9	1	0.95	1	1
TCGA-UCEC-TP	1	0.99	1	1	1
accuracy	0.99	0.99	0.99		
macro avg	0.98	0.97	0.97		
weighted avg	0.99	0.99	0.99		

Precision, recall, f1-score, specify, and AUC, for ensemble classifier using 150 interacting miRNAs, as a feature set from the RFE, method of feature selection.

**TABLE 7:** Performance of Ensemble classifier with random forest miRNA features.

Response class	RF_precision	RF_recall	RF_f1-score	RF_Specificity	RF_AUC
TCGA-BLCA-NT	1	0.83	0.91	1	1
TCGA-BLCA-TP	0.99	1	1	1	1
TCGA-BRCA-NT	1	0.84	0.91	1	0.99
TCGA-BRCA-TP	0.98	1	0.99	1	1
TCGA-ESCA-TP	1	1	1	1	1
TCGA-HNSC-NT	1	0.92	0.96	1	1
TCGA-HNSC-TP	0.99	1	1	1	1
TCGA-KICH-NT	1	1	1	1	1
TCGA-KICH-TP	1	1	1	1	1
TCGA-KIRC-NT	1	1	1	1	1
TCGA-KIRC-TP	1	1	1	1	1
TCGA-KIRP-NT	1	0.9	0.95	1	1
TCGA-KIRP-TP	0.99	1	0.99	1	1
TCGA-LIHC-NT	0.94	1	0.97	1	1
TCGA-LIHC-TP	1	0.99	1	1	1
TCGA-LUAD-NT	0.93	0.93	0.93	1	1
TCGA-LUAD-TP	0.99	0.99	0.99	1	1
TCGA-LUSC-NT	1	1	1	1	1
TCGA-LUSC-TP	1	1	1	1	1
TCGA-PRAD-NT	0.93	0.93	0.93	1	1
TCGA-PRAD-TP	0.99	0.99	0.99	1	1
TCGA-STAD-NT	0.92	0.86	0.89	1	1
TCGA-STAD-TP	0.99	0.99	0.99	1	1
TCGA-THCA-NT	0.94	0.94	0.94	1	1
TCGA-THCA-TP	0.99	0.99	0.99	1	1
TCGA-UCEC-NT	1	0.89	0.94	1	1
TCGA-UCEC-TP	0.99	1	1	1	1
accuracy	0.99	0.99	0.99		
macro avg	0.98	0.96	0.97		
weighted avg	0.99	0.99	0.99		

Precision, recall, f1-score, specify, and AUC, for ensemble classifier using 298 interacting miRNAs, as a feature set from the RF, method of feature selection.

**TABLE 8:** Performance of Ensemble classifier with Boruta miRNA features.

Response class	Boruta_precision	Boruta_recall	Boruta_f1-score	Boruta_Specificity	Boruta_AUC
TCGA-BLCA-NT	1	0.67	0.8	1	1
TCGA-BLCA-TP	0.98	1	0.99	1	1
TCGA-BRCA-NT	0.96	0.87	0.92	1	0.99
TCGA-BRCA-TP	0.99	1	0.99	1	1
TCGA-ESCA-TP	1	1	1	1	1
TCGA-HNSC-NT	1	1	1	1	1
TCGA-HNSC-TP	1	1	1	1	1
TCGA-KICH-NT	1	1	1	1	1
TCGA-KICH-TP	1	1	1	1	1
TCGA-KIRC-NT	1	1	1	1	1
TCGA-KIRC-TP	1	1	1	1	1
TCGA-KIRP-NT	1	0.9	0.95	1	1
TCGA-KIRP-TP	0.99	1	0.99	1	1
TCGA-LIHC-NT	0.93	0.93	0.93	1	1
TCGA-LIHC-TP	0.99	0.99	0.99	1	1
TCGA-LUAD-NT	0.93	0.93	0.93	1	1
TCGA-LUAD-TP	0.99	0.99	0.99	1	1
TCGA-LUSC-NT	1	0.93	0.96	1	1
TCGA-LUSC-TP	0.99	1	1	1	1
TCGA-PRAD-NT	0.93	0.93	0.93	1	1
TCGA-PRAD-TP	0.99	0.99	0.99	1	1
TCGA-STAD-NT	0.92	0.79	0.85	1	1
TCGA-STAD-TP	0.98	0.99	0.99	1	1
TCGA-THCA-NT	0.94	0.89	0.91	1	1
TCGA-THCA-TP	0.99	0.99	0.99	1	1
TCGA-UCEC-NT	1	1	1	1	1
TCGA-UCEC-TP	1	1	1	1	1
accuracy	0.99	0.99	0.99		
macro avg	0.98	0.96	0.97		
weighted avg	0.99	0.99	0.99		

Precision, recall, f1-score, specify, and AUC, for ensemble classifier using 530 interacting miRNAs, as a feature set from the Boruta method of feature selection.

**TABLE 9:** Performance of Ensemble classifier with LDA miRNA features.

Response class	LDA_precision	LDA_recall	LDA_f1-score	LDA_Specificity	LDA_AUC
TCGA-BLCA-NT	1	0.83	0.91	1	1
TCGA-BLCA-TP	0.99	1	1	1	1
TCGA-BRCA-NT	0.96	0.84	0.9	1	0.99
TCGA-BRCA-TP	0.98	1	0.99	1	1
TCGA-ESCA-TP	1	1	1	1	1
TCGA-HNSC-NT	1	0.92	0.96	s1	1
TCGA-HNSC-TP	0.99	1	1	1	1
TCGA-KICH-NT	1	1	1	1	1
TCGA-KICH-TP	1	1	1	1	1
TCGA-KIRC-NT	1	1	1	1	1
TCGA-KIRC-TP	1	1	1	1	1
TCGA-KIRP-NT	1	0.9	0.95	1	1
TCGA-KIRP-TP	0.99	1	0.99	1	1
TCGA-LIHC-NT	0.92	0.8	0.86	1	1
TCGA-LIHC-TP	0.97	0.99	0.98	1	1
TCGA-LUAD-NT	1	0.93	0.96	1	1
TCGA-LUAD-TP	0.99	1	1	1	1
TCGA-LUSC-NT	1	1	1	1	1
TCGA-LUSC-TP	1	1	1	1	1
TCGA-PRAD-NT	1	0.87	0.93	1	1
TCGA-PRAD-TP	0.99	1	0.99	1	1
TCGA-STAD-NT	1	0.86	0.92	1	1
TCGA-STAD-TP	0.99	1	0.99	1	1
TCGA-THCA-NT	0.94	0.89	0.91	1	1
TCGA-THCA-TP	0.99	0.99	0.99	1	1
TCGA-UCEC-NT	1	1	1	1	1
TCGA-UCEC-TP	1	1	1	1	1
accuracy	0.99	0.99	0.99		
macro avg	0.99	0.96	0.97		
weighted avg	0.99	0.99	0.99		

Precision, recall, f1-score, specify, and AUC, for ensemble classifier using 352 interacting miRNAs, as a feature set from the LDA, method of feature selection.

**FIGURE 3:**
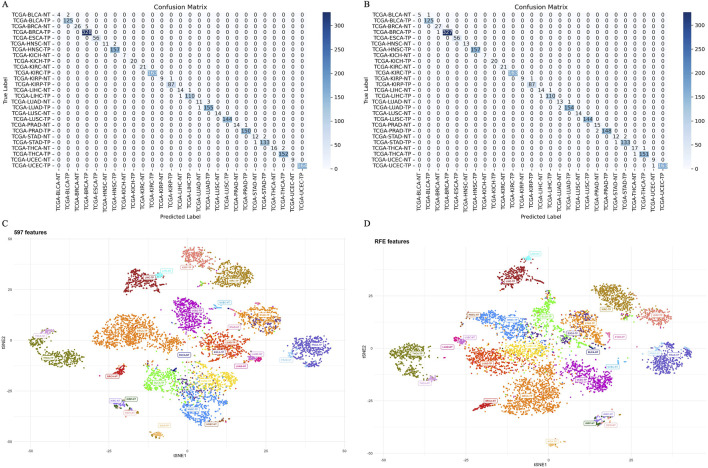
Confusion matrix and t-SNE plot, depicting models’ separability **(A)** Confusion matrix of the ensemble model trained with 597 miRNA features. **(B)** Confusion matrix of the ensemble model trained with RFE (150 miRNA) features. **(C)** t-SNE projection of 597 miRNAs across 14 cancer types, illustrating models’ separability. **(D)** t-SNE projection of 150 miRNAs (RFE feature set) across 14 cancer types, illustrating model separability.

The ensemble model using 597 miRNA features performed below 80% for the BLCA-NT (recall = 67%) and LUAD-NT (recall = 79%) classes. Similarly, the Boruta feature set model (530 miRNAs) also performed below 80% for the BLCA-NT class (recall = 67%) and the stomach adenocarcinoma (STAD-NT) class (recall = 79%). In contrast, the RFE feature set (150 miRNAs) showed superior performance for several classes, including breast invasive carcinoma (BRCA-NT/TP), esophageal carcinoma (ESCA-TP), head and neck squamous cell carcinoma (HNSC-NT/TP), kidney chromophobe (KICH-NT/TP), kidney renal clear cell carcinoma (KIRC-NT/TP), kidney renal papillary cell carcinoma (KIRP-NT/TP) as compared to the RF (298 miRNAs) and LDA model (352 miRNAs). The RF model performed better in the liver hepatocellular carcinoma (LIHC-NT) class than the RFE and LDA models. For the LUAD-NT/TP, STAD-NT/TP, and uterine corpus endometrial carcinoma (UCEC-NT/TP) classes, the LDA-based model outperformed the RFE and RF feature set-based models. The RFE feature set model demonstrated similar performance to the RF feature set model for THCA-NT, however it outperformed the RF model for the UCEC-NT class. A bar plot comparing ensemble model performance using the RFE, RF, and LDA feature sets is shown in Figures 2A–E. The confusion matrix plot (Figures 3A, B; Supplementary Figures S2A–C) highlights the ensemble classifier’s true classification counts per cancer type across all feature sets. Additionally, t-SNE projections of the 597-miRNA feature set and the RFE feature set (150 miRNAs) are shown in Figures 3C, D, respectively.

### 3.5 Feature importance analysis and survival outcomes

**FIGURE 4:**
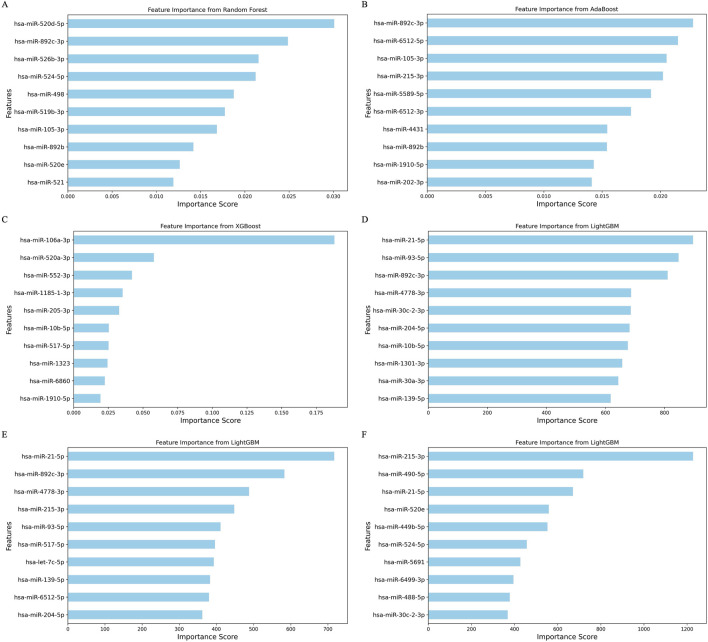
Bar plot depicting feature importance from the 4 ML methods. **(A)** Random forest model with the random forest-based feature set. **(B)** AdaBoost model with the Boruta feature set. **(C)** XGBoost model with the random forest feature set. **(D)** LightGBM model with the RFE feature set. **(E)** LightGBM model with the random forest feature set. **(F)** LightGBM model with the LDA feature set.

In our feature importance analysis, we evaluated the contributions of individual miRNAs to the predictive models. The most important features, according to the RF, AdaBoost, XGBoost, and LightGBM models, respectively, highlight the topmost impactful miRNA for each model in Figures 4A–E. Notably, several miRNAs, including *miR-520d-5p, miR-520a-3p, miR-520e, miR-892c-3p, miR-892b, miR-105-3p, miR-215-3p, miR-10b-5p, miR-139-5p, miR-21-5p, miR-93-5p, miR-4778-3p, miR-30c-2-3p,* and *miR-204-5p,* emerged as common top features across models, suggesting their significant role in cancer progression. The top features for each trained model, along with their interacting genes and lncRNAs from the network, are highlighted in Supplementary Table S7.

**FIGURE 5:**
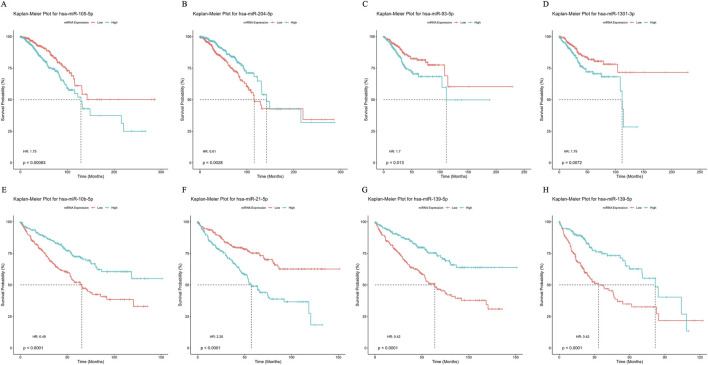
Survival curve depicting prognostic capabilities of miRNA biomarkers using Kaplan-Meier plot for **(A–B)** Breast Invasive Carcinoma (BRCA) **(C–D)** Uterine Corpus Endometrial Carcinoma (UCEC). **(E–G)** Kidney Renal Papillary Cell Carcinoma (KIRC) and **(H)** Liver Hepatocellular Carcinoma (LIHC).

The survival analysis results further emphasized the prognostic potential of these miRNAs in various cancer types. For example, higher expression of *miR-204-5p* in BRCA correlated with improved survival outcomes (HR < 1; p < 0.0001), whereas lower expression of *miR-105-5p* was linked to poorer prognosis (HR > 1; p < 0.0001) for patients (Figures 5A, B). In UCEC, patients with elevated miR-93-5p and miR-1301-3p expression levels exhibited a median survival of approximately 120 months (HR > 1), indicating poor prognostic markers (Figures 5C, D). Similarly, in KIRC, high expression of *miR-10b-5p* and *miR-139-5p* correlated with better survival than the low expression group (HR < 1; p < 0.0001), while elevated *miR-21-5p* levels predicted worse survival outcomes (HR > 1; p < 0.0001) (Figures 5E, F). In LIHC, high *miR-139-5p* expression significantly improved survival outcomes compared to low expression levels, as indicated by the statistical significance (p < 0.0001) and the hazard ratio (HR = 0.42) (Figure 5G). Collectively, these results highlight the prognostic relevance of these miRNAs, suggesting their potential as cancer prognostic biomarkers.

**FIGURE 6:**
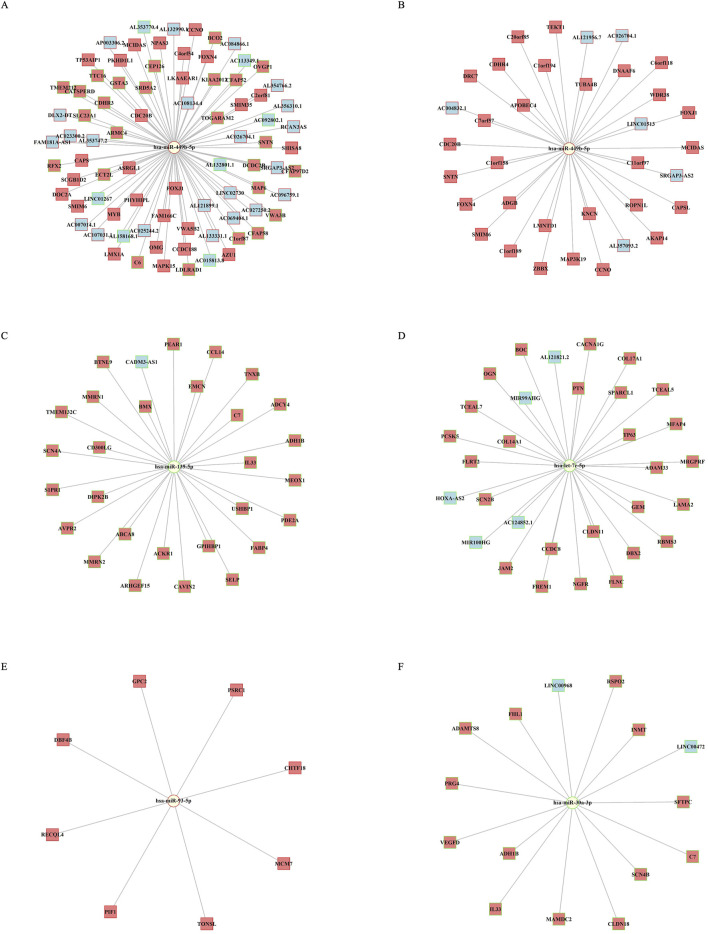
miRNA-mRNA-lncRNA network visualization. **(A)** Network of miR-499b-5p in UCEC. **(B–D)** Network of miR-499b-5p, miR-139-5p, and let-7c-5p in BRCA. **(E–F)** Network in LUAD for miR-93-5p, miR-30a-3p. The log2 fold change values highlight node borders: green for downregulated and red for upregulated miRNAs, mRNAs, and lncRNAs. Node shapes represent miRNAs (circles), protein-coding genes (squares), and lncRNAs (c-shaped squares).

The interaction networks of top miRNA features are plotted for UCEC, BRCA, and LUAD (Figures 6A–C). In UCEC and BRCA, *miR-499bc-5p* demonstrated the highest degree of centrality, connecting 81 nodes in UCEC and 35 in BRCA. In UCEC, miRNA interacted with both upregulated and downregulated genes, while in BRCA, its network connections were confined to upregulated genes (Figures 6A, B). The networks in all three cancers were sparse with miRNAs linked to multiple genes. *MiR-139-5p* and *let-7c-5p* are both downregulated and were associated with the expression of downregulated genes, with degree centralities of 29 and 35, respectively. In the LUAD network, *miR-93-5p* had a degree centrality of 8, whereas *miR-30a-3p* had a degree centrality of 15, indicating similar regulatory patterns (Figure 6C). These interactions further emphasize the important co-regulatory roles of miRNAs in cancer progression and survival.

### 3.6 Overlap of predictive miRNAs with literature compendium, clinical trials, and drug target associations

**FIGURE 7:**
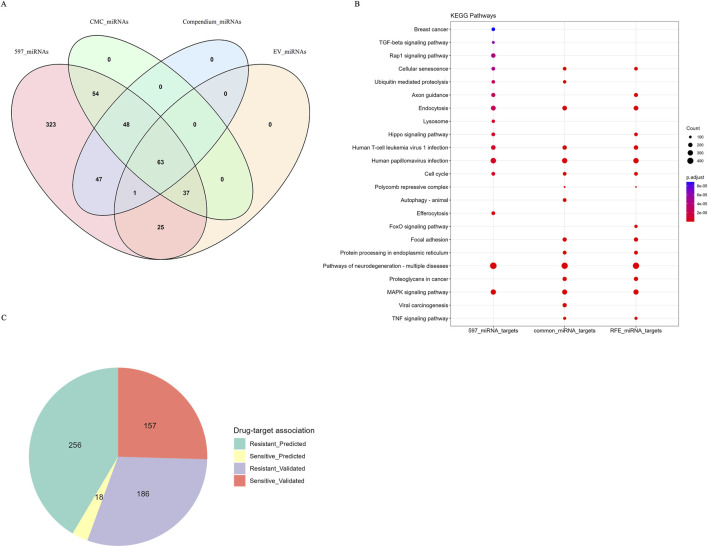
Overlapping miRNAs with miRNA compendium, CMC db, EV miRNAs and noncoRNA db and KEGG enrichment analysis. **(A)** Venn diagram depicting overlapping miRNAs features with miRNA compendium and CMC database and EV miRNAs **(B)** Dot plot depicting KEGG pathway enrichment analysis. **(C)** Pie chart depicting miRNAs sensitive to drugs and the experimental evidence of drug-target associations from noncoRNA db.

Our findings revealed a substantial overlap between the predictive miRNA biomarkers identified in this study and those reported in existing literature, ongoing clinical trials, and the CMC, underscoring their relevance in cancer-related research (Figure 7A). Specifically, a total of 159 predictive miRNAs matched entries in the manually curated miRNA literature-derived compendium (Supplementary Table S8). Of these, 126 miRNAs have been reported as being EV in various cancers. Furthermore, 202 miRNAs from the CMC list corresponded to our identified features. Across the four miRNA datasets, including interacting miRNAs (597 miRNAs), literature-derived miRNAs (miRNA isoforms and EV miRNAs), and miRNAs from the CMC list, 63 miRNAs were common across all datasets, highlighting their significant relevance in cancer progression.

Several specific miRNAs identified in our analysis have well-established associations with cancer. For example, *miR-106a-3p/5p* is recognized for targeting *PTEN* in prostate cancer, while the *miR-125* cluster significantly influences the same type of cancer by targeting p53 inhibitors (Wu et al., 2024). Likewise, both the *miR-200* and *miR-204-3p/5p* clusters are associated with thyroid cancer, and *miR-21-3p/5p* has connections to both lung and thyroid cancers (Cabané et al., 2024; Carrà et al., 2024). The common miRNAs found in EVs play significant roles in cancer progression by influencing the growth and proliferation of tumors. Specifically, the *miR-155* cluster and the *miR-221* cluster are associated with renal cell carcinoma and breast cancer, respectively (Das et al., 2019; Liwei et al., 2021; Parashar et al., 2024). Additionally, the *miR-181c,* the *miR-200* cluster, and the *miR-221* cluster have been implicated in breast cancer metastasis (Das et al., 2019; Parashar et al., 2024; Tominaga et al., 2015; Le et al., 2014). In terms of angiogenesis, *miR-181b-5p* in esophageal squamous cell carcinoma, as well as *miR-143* and *miR-145* clusters in lung cancers, and *miR-15a* and *miR-181b* in renal carcinomas, all influence this vital process (Parashar et al., 2024; Wang et al., 2020; Lawson et al., 2017; Lopatina et al., 2019).

Furthermore, *miR-31-5p* and *miR-222-3p* from renal cell carcinoma and non-small cell lung cancer are involved in chemotherapy resistance (Parashar et al., 2024; He et al., 2020; Wei et al., 2017). Conversely, *miR-503* in breast cancer cells has been shown to enhance sensitivity to chemotherapy by disrupting cell proliferation and invasion (Parashar et al., 2024; Bovy et al., 2015). These miRNAs are highlighted as crucial elements in our interaction network, as shown in the Supplementary Table S7.

Our analysis identified a considerable number of miRNAs that are currently being investigated in clinical trials for cancer therapies, as shown in Table 10, underscoring their translational potential (Supplementary Table S9). We also evaluated the overlap between the identified miRNA features and those in the noncoRNA db, as shown in the pie chart (Figure 7C) revealed associations with drug targets. Among the 617 miRNAs associated with drug targets, we found 186 miRNAs in validated associations that were resistant to cancer therapies, while 157 miRNAs were linked to treatment sensitivity. Overall, our analysis revealed 256 predicted resistance associations and 18 predicted sensitivity associations. A complete overview of the drug-target associations is provided in Supplementary Table S10.

**TABLE 10:** miRNAs in ongoing clinical trial studies.

miRNAs in clinical trial	Cancer
hsa-miR-10b-3p	Glioblastoma
hsa-miR-10b-5p	Glioblastoma
hsa-miR-1307-3p	pancreatic cancer
hsa-miR-1307-5p	pancreatic cancer
hsa-miR-146a-5p	lung cancer
hsa-miR-155-3p	lymphoma, breast cancer
hsa-miR-155-5p	lymphoma, breast cancer
hsa-miR-16-1-3p	lung cancer
hsa-miR-16-2-3p	lung cancer
hsa-miR-18a-3p	breast cancer
hsa-miR-18a-5p	breast cancer
hsa-miR-193a-3p	advanced solid tumors
hsa-miR-211-5p	ovarian cancer
hsa-miR-218-5p	lung cancer
hsa-miR-22-3p	lung cancer
hsa-miR-29b-2-5p	lung cancer
hsa-miR-29b-3p	lung cancer
hsa-miR-34a-5p	Renal cell carcinoma, non small cell lung cancer (NSCLC), liver cancer
hsa-miR-7-2-3p	lung cancer, gastric cancer
hsa-miR-7-5p	lung cancer, gastric cancer
hsa-miR-9-3p	Lung cancer
hsa-miR-9-5p	lung cancer

### 3.7 Functional enrichment analysis

To explore the biological roles of the 597 interacting miRNAs identified in our study, we conducted GO and KEGG pathway enrichment analyses on their experimentally validated targets. The findings from GO analysis revealed significant enrichment in biological processes critical for cancer progression, including cellular adhesion, differentiation, organelle localization, embryonic organ development, renal and kidney development, T-cell differentiation, and DNA replication (Supplementary Figure S3). Further, KEGG pathway enrichment analysis was performed on the targets of all 597 miRNAs, the 150 miRNA features selected by the RFE method, and the 63 miRNAs shared across the 597 miRNAs, the literature compendium, EV miRNAs, and CMC miRNAs (Figure 7B). The top enriched pathways resulting from the above-mentioned analysis included cellular senescence, Hippo signaling, *FoxO* signaling, *MAPK* signaling, *TNF* signaling, and pathways related to Human Papillomavirus (HPV) infections. These enriched pathways highlight the central role of miRNAs in key signaling cascades implicated in cancer biology. Detailed data from the GO and KEGG enrichment analyses are presented in Supplementary Tables S11–14. miRNA interactions common to validated and predicted interactions for 597 miRNAs, extracted from the databases such as miRTarBase, TarBase, miRecords, Pictar, and Diana, -obtained from multiMiR R package were shown in Supplementary Tables S15, 16.

### 3.8 Classification performance of mRNA/lncRNA classifiers

The lightGBM method was used to classify 14 cancer types using interacting mRNAs and lncRNAs as the features. The classification results for mRNA/lncRNA-based ML models are shown in Supplementary Tables S17, 18. The models had an overall accuracy of 98%–99% in both cases. However, they did not perform as well when classifying some cancer types. For the mRNA models, all three models showed lower sensitivity for normal samples in these classes: BLCA-NT, HNSC-NT, LUAD-NT, PRAD-NT, STAD-NT, and UCEC-NT. The recall values for these classes were below 80%. Additionally, the F1-score was lower than 80% for the following classes: BLCA-NT, HNSC-NT, PRAD-NT, and STAD-NT. Similarly, the lncRNA feature models had lower precision, in the case of KICH-NT and BLCA-NT, lower recall/sensitivity, and F1 -score for BLCA-NT, PRAD-NT, STAD-NT, and UCEC-NT (<80%). The same LightGBM models trained on miRNA features performed better in classifying these normal samples than the mRNA or lncRNA feature sets.

## 4 Discussion

The current study aimed to identify a minimal set of miRNA biomarkers capable of distinguishing primary cancer types based on their TOO while considering the complex interactions among miRNAs, mRNAs, and lncRNAs. Correlation-based networks infer regulatory relationships by analyzing co-expression profiles, which capture molecular interactions in cancer progression (Zheng et al., 2020; Yang et al., 2022), rather than relying on sequence analysis of RNA interactions (Adinolfi et al., 2019; Bheemireddy et al., 2022). The availability of extensive and standardized expression datasets from the TCGA makes correlation-based methods particularly effective for constructing robust and biologically meaningful co-expression networks in cancer studies. By integrating these interactions, our approach provides a more biologically relevant and robust set of miRNA signatures, enhancing the potential for early cancer detection.

Our ensemble learning framework, combining Random Forest, AdaBoost, XGBoost, and LightGBM, achieved an impressive 99% accuracy in classifying 14 distinct cancer types based on TOO. We visualized the clustering of cancer samples according to tissue types using t-SNE plots (Figures 3C, D), which demonstrated higher discrimination power. Despite achieving high overall accuracy in TOO prediction, our study revealed some variations in model performance for specific cancer types (Figures 2A–E). These variations can be attributed to factors such as molecular complexity. Gastric cancer, specifically STAD, is often challenging to classify in molecular studies (Cao et al., 2022). In contrast, cancers with well-characterized molecular profiles, such as BRCA and lung cancer (e.g., LUSC) (Koboldt et al., 2012; Thennavan et al., 2021; Hammerman et al., 2012), exhibited higher and more consistent accuracy across all models and feature selection methods. This consistent performance for BRCA and lung cancers highlights the critical role of distinct molecular signatures in improving classification accuracy. These results suggest the need for tailored optimization strategies to enhance classification outcomes, especially for complex and heterogeneous cancer types like gastric cancer.

While previous studies have demonstrated promising results in tumor origin classification, they often face limitations in accurately classifying specific cancer types or lack comprehensive biological validation. For instance, Raghu et al. (2024) (Raghu et al., 2024) achieved a high accuracy (97%) for tumor origin detection, however, their method struggled with cancers like uterine (77% with decision tree) and esophagus (33.3% with decision tree and 83% with deep learning) cancers, highlighting limitations in certain cancer classifications. Similarly, Tang et al. (2018) (Tang et al., 2018) used miRNA and DNA methylation markers, achieving ∼91% and ∼96% accuracy, respectively, but relying solely on single-layer data. Another comparative study demonstrates that DNA methylation profiles, particularly when analyzed using LASSO and neural network models, offer the highest predictive accuracy, ∼97.77% for tumor tissue origin detection compared to mRNA, microRNA, and lncRNA expression profiles​ (Feng and Wang, 2024). Lopez-Rincon et al. (2020) focused on comprehensive feature selection with ensemble methods providing minimal miRNAs for classification. A study by Matsuzaki et al. (2023) on serum miRNomes for predicting the TOO in early-stage cancers showed an 88% accuracy across all stages (Matsuzaki et al., 2023). Unlike the studies mentioned earlier, our research integrates miRNA-mRNA-lncRNA interactions identified from co-expression networks, crucial for understanding cancer initiation and pathways, as demonstrated in other cancer studies (Dong et al., 2020; Zheng et al., 2020; Naghsh-Nilchi et al., 2022; Gao et al., 2021). Our approach includes thorough *in silico* validation using CMC, analysis of survival markers, assessment of drug sensitivity, and relevance to clinical trials. This multi-layered approach provides more biologically relevant insights, positioning our study as a more comprehensive tool for cancer classification and therapeutic planning.

Our methodological approach demonstrated the power of Artificial Intelligence (AI) in complex multiclass classification. Our feature selection process identified several important miRNAs. These include miR-21-5p, miR-93-5p, and miR-10b-5p (Qian et al., 2024; Yan et al., 2021; Pan et al., 2021). These miRNAs are linked to critical tumorigenic processes. These processes include immune modulation, epithelial-mesenchymal transition, angiogenesis, and chemoresistance (Pavlíková et al., 2022). We also conducted an *in silico* validation. This validation revealed overlaps between these miRNA features and drug-target associations. This highlights their dual role in regulating drug sensitivity (Seyhan, 2024; Si et al., 2019) and chemoresistance (Pavlíková et al., 2022). Overall, these miRNAs have an influence on essential processes. These processes include apoptosis, immune response, and therapy resistance. This underscores their potential to guide personalized cancer treatments (Mishra et al., 2016). Functional enrichment analysis, including GO and pathway analysis of miRNA targets, uncovered significant KEGG pathways and GO terms. These terms are associated with both normal biological processes (e.g., embryonic organ development, the establishment of organelle localization, DNA replication), tissue differentiation (e.g., mononuclear cell differentiation, renal system development), and cancer-specific mechanisms involved in cancer development (e.g., T cell differentiation). As highlighted in previous studies (Khatun et al., 2024; Khatun et al., 2021), our study provides an intricate association between HPV and gynecological cancers by incorporating advanced machine learning approaches and rigorous *in silico* validation methods. Our findings emphasize the role of various cellular mechanisms in cancer development and progression, along with key cancer pathways (Figure 7B), which are consistent with previous studies (Xing et al., 2016; Andrés-León et al., 2017).

The top miRNA features identified by our ML models (Supplementary Table S7) were associated with patient prognosis, with several of those implicated in ongoing clinical trials, consistent with findings from previous studies (Seyhan, 2024; Kim and Croce, 2023; Hanna et al., 2019). For instance, RNA-based therapies targeting *miR-21-5p* have addressed immune infiltration and poor prognosis in KIRC (Rhim et al., 2022; Jenike and Halushka, 2021; Wang et al., 2022). *miR-93-5p* enhances radiosensitivity by increasing apoptosis in breast cancer (Pan et al., 2021) while promoting tumor progression in the bladder (Yuan et al., 2023) and esophageal carcinoma cells (Xu, 2019). *miR-204-5p* acts as a tumor suppressor in laryngeal squamous cell carcinoma (LSCC) (Gao et al., 2017; Fan et al., 2023), targets anti-apoptotic protein BCL2 in prostate cancer (PCa) (Lin et al., 2017) and serves as an early diagnostic biomarker in endometrial cancer (EC) (Wu et al., 2022). *miR-10b-5p* regulates gastric cancer (GC) fibroblast interactions via the *TGFβ* signaling pathway (Yan et al., 2021), while *miR-1301-3p* is a potential therapeutic target for thyroid papillary carcinoma (Qiao et al., 2021), gastric cancer (Luo et al., 2021), and endometrial cancer (Lu et al., 2021). Overall, these findings highlight the multifaceted role of miRNAs in distinguishing TOO as diagnostic biomarkers and potential therapeutic targets, offering unifying translational tools for leveraging circulating miRNAs for personalized medicine across pan-cancers/various cancer types.

### 4.1 Strengths and limitations

Our comprehensive study has several notable strengths. The inclusion of 14 cancer types ensures broader applicability and cost-effectiveness. This was complemented by TCGA data, which provided a larger sample size, enhancing the reliability and generalizability of our findings. The integration of advanced ML models with biologically informed feature selection and a multi-validation approach, comprising functional enrichment analyses and clinical trial associations, collectively enhances the robustness of our analytical framework. Furthermore, the identification of key miRNAs with significant diagnostic potential emphasizes the translational relevance of this study. By accounting for complex molecular interactions and addressing gaps in existing studies, our study offers improved diagnostic precision.

Despite these potential strengths, certain limitations persist. First, the complexity of miRNA interaction networks poses challenges for experimental validation. Our study relied exclusively on TCGA data, which, while comprehensive, may not fully represent the heterogeneity of cancer subtypes, particularly in rare cases. Additionally, a limitation of this study is the lack of detailed subtype information and metastatic samples, as our analysis was restricted to TCGA-derived primary tumor datasets. Future work will aim to incorporate these aspects to enhance the resolution and applicability of the classification model. Incorporating multiple clinical cohorts and more comprehensive clinical data could further improve our understanding of the role of these miRNA biomarkers in cancer. Finally, while the use of solid tissue samples offers valuable insights, their inherent heterogeneity limits the clinical translation of miRNA biomarkers. Future studies incorporating liquid biopsy data and multi-omics approaches could enhance the translational potential of our findings.

## 5 Conclusion and future research

In summary, our study demonstrated the potential of integrating biologically relevant miRNA features with advanced ML approaches to achieve high accuracy in TOO prediction. Through *in silico* validation, including functional enrichment analysis, survival analysis, clinical trial associations, and drug sensitivity correlations, we highlighted the biological significance and therapeutic potential of the identified miRNAs. These findings emphasize the importance of integrating computational approaches with biological insights to improve the robustness of cancer diagnostics and treatment. Although the predictive power is promising, further experimental validation is warranted to confirm the clinical relevance of these miRNAs, ultimately advancing precision oncology and improving patient care. Future studies should explore the application of miRNAs in precisely classifying cancer subtypes and accurately determining the origins of metastatic tumors using samples from solid tissues or bodily fluids.

## Data Availability

The TCGA data used in the study is publicly available at https://portal.gdc.cancer.gov/, and the miRNA compendium created from the literature (Supplementary Table S1) and all supplementary Tables are available at Zenodo (https://doi.org/10.5281/zenodo.15094619). All R codes and Python codes used in the analysis and ML are available through the GitHub repository: https://github.com/ankita16lawarde/ML_miRNA.
