# Supplementary material for: Tumor tissue-of-origin classification using miRNA-mRNA-lncRNA interaction networks and machine learning methods
Source: Front Bioinform. 2025 May 6;5:1571476. doi: 10.3389/fbinf.2025.1571476 (PMC12088952; doi:10.3389/fbinf.2025.1571476)
Supplement: Supplementary file 1 [file DataSheet1.docx]

**Tumor Tissue-of-Origin Classification Using miRNA-mRNA-lncRNA Interaction Networks and Machine Learning Methods**

Ankita Lawarde^a,b^, Masuma Khatun^c^, Prakash Lingasamy^a,b^, Andres Salumets^a,b,d*^, Vijayachitra Modhukur ^a,b*^

**Affiliations**: ^a^Department of Obstetrics and Gynecology, Institute of Clinical Medicine, University of Tartu, 50406 Tartu, Estonia; ^b^ Celvia CC AS, 50411 Tartu, Estonia; ^c^ Department of Obstetrics and Gynecology, University of Helsinki and Helsinki University Hospital, Haartmaninkatu 8, 00290 Helsinki, Finland; ^d^Division of Obstetrics and Gynecology, Department of Clinical Science, Intervention and Technology (CLINTEC), Karolinska Institute, and Karolinska University Hospital, Stockholm, Sweden.

* Indicate corresponding authors

**Corresponding authors:**

(1) Andres Salumets, Division of Obstetrics and Gynecology, Department of Clinical Science, Intervention and Technology (CLINTEC), Karolinska Institute, and Karolinska University Hospital, 14152, Stockholm, Sweden. E-mail: andres.salumets@ki.se. https://orcid.org/0000-0002-1251-8160

(2) Vijayachitra Modhukur, Department of Obstetrics and Gynecology, Institute of Clinical Medicine, University of Tartu, 50406 Tartu, Estonia. E-mail: modhukur@ut.ee. https://orcid.org/0000-0002-7123-9903

These authors jointly supervised this work (last authors): Andres Salumets, Vijayachitra Modhukur

**This file includes:**

Supplementary figures

**Figure S1**: Venn diagram of five feature sets

**Figure S2:** Confusion matrix of ensemble models

**Figure S3:** Dot plot of GO enrichment analysis of 597 miRNA targets.

**Legends of supplementary tables**

**Supplementary Figure 1:**


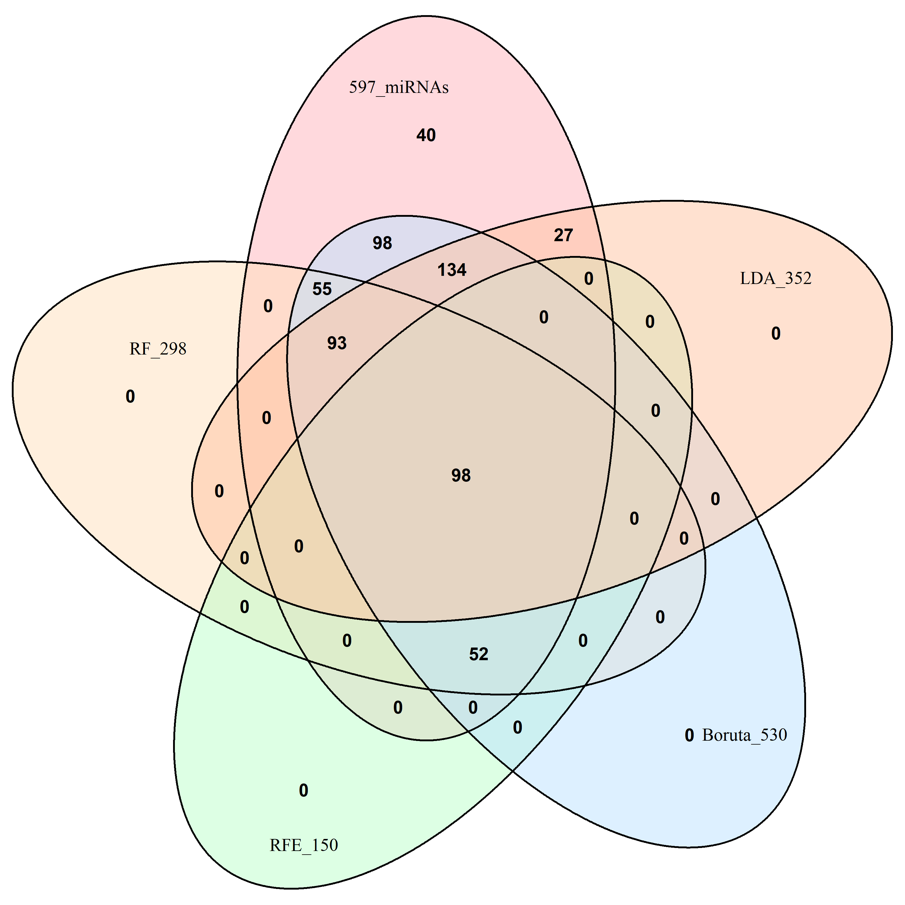


**Figure S1**: Venn diagram of five feature sets. An overlap between 597 miRNA features from network analysis, 150 miRNAs from Recursive elimination features (RFE), 298 miRNAs from Random Forest (RF) feature set, 530 miRNAs from the Boruta feature set, and 352 miRNAs from the Linear discriminant analysis (LDA) feature set.

**Supplementary Figure 2:**


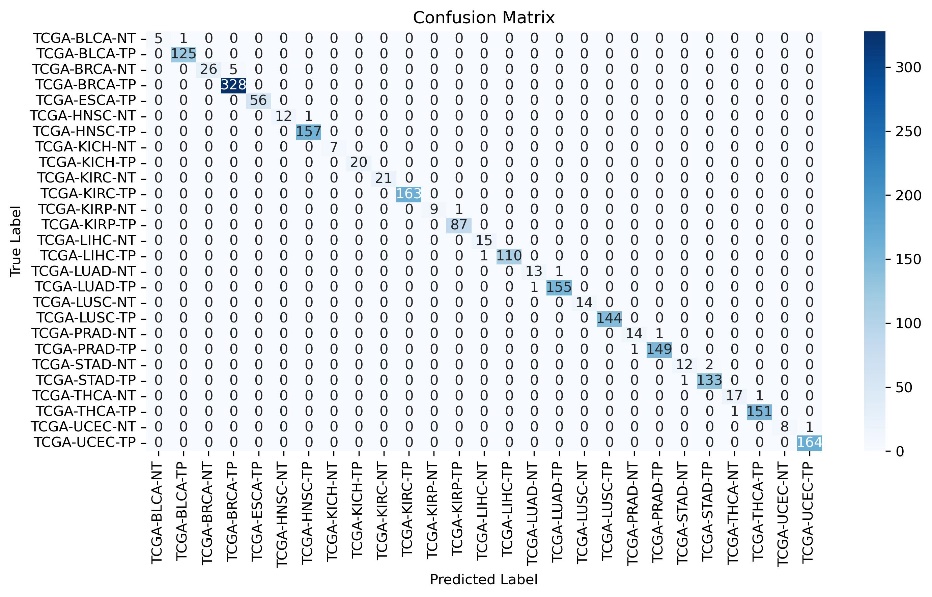


**A**


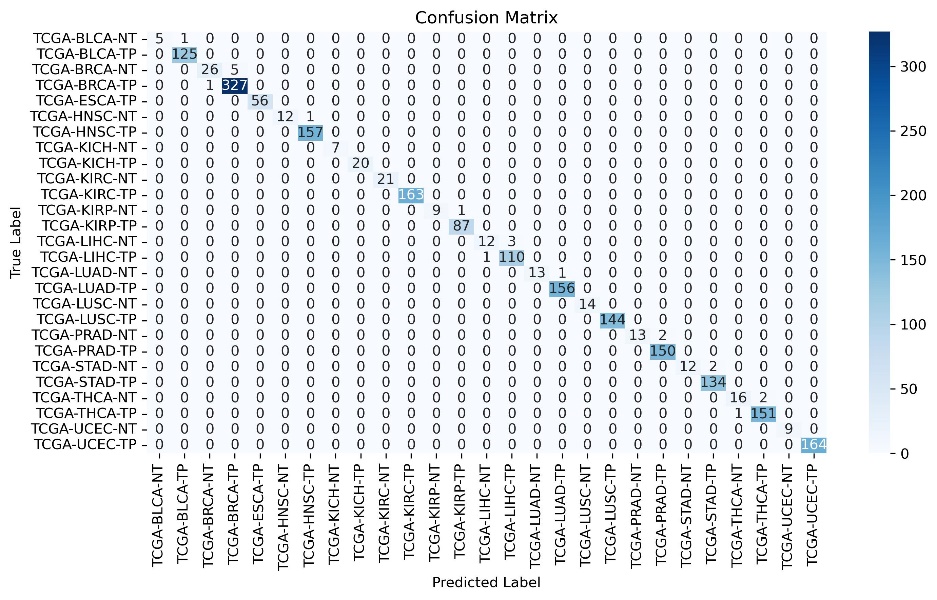


**C**


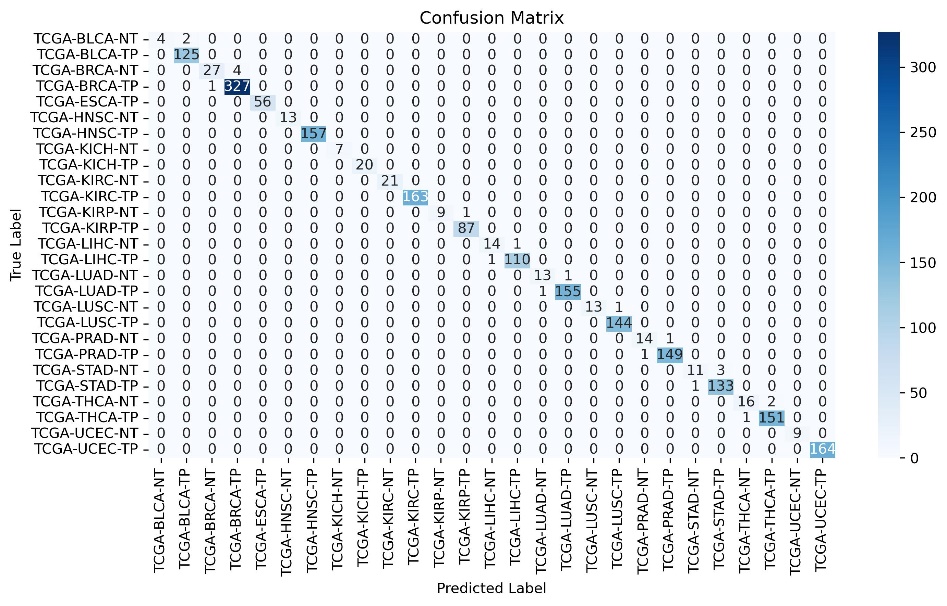


**B**

**Figure S2:** Confusion matrix of ensemble models trained on (A) Random Forest (RF) feature set of 298 miRNAs, (B) Boruta feature set of 530 miRNAs, and (C) Linear discriminant analysis (LDA) of 352 miRNAs, respectively.


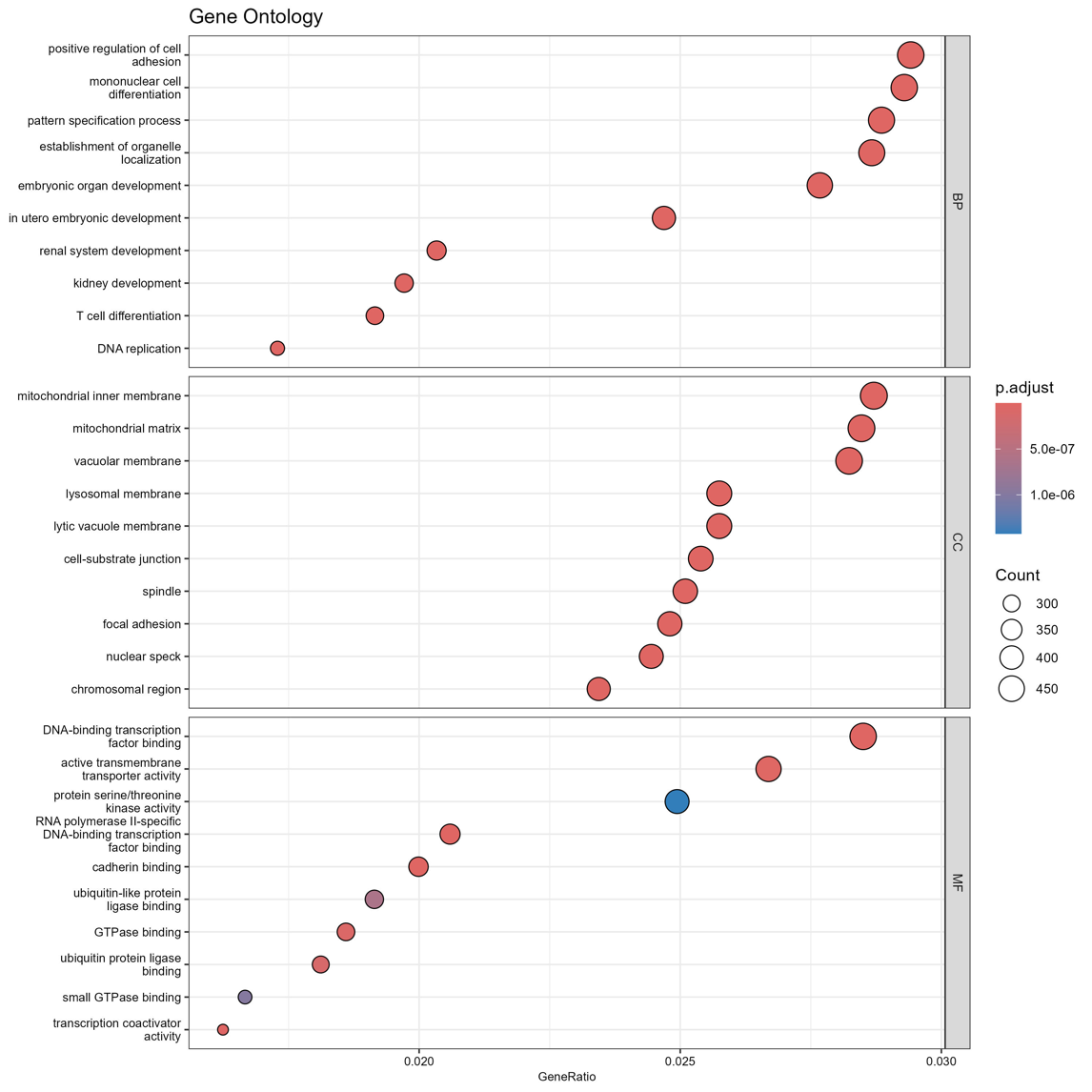
**Supplementary Figure 3:**

**Figure S3:** Dot plot of GO enrichment analysis of 597 miRNA targets. The top 10 enriched terms Biological Processes (BP), Cellular components (CC), and Molecular function (MF) are shown. Range P-adjust values are shown with the color bar, and the size of the dot corresponds to the number of genes present in each identified term.

**Supplementary data Legends:**

**Supplementary Table 1:** Edge table for 14 cancer types miRNA-mRNA-lncRNA interaction network and degree centrality of miRNAs.

**Supplementary Table 2:** Table of miRNA list from each feature selection method. 597 interacting miRNAs, 298 miRNAs from RF, 150 miRNAs from RFE, 530 miRNAs from Boruta and 352 miRNAs from LDA method.

**Supplementary Table 3:** Table of RF method performance with each feature set. The table contains the precision, recall/sensitivity, F1-score, specificity, and AUC for RF with five feature sets.

**Supplementary Table 4:** Table of AdaBoost method performance with each feature set. The table contains the precision, recall/sensitivity, F1-score, specificity, and AUC for AdaBoost with five feature sets.

**Supplementary Table 5:** Table of XGBoost method performance with each feature set. The table contains the precision, recall/sensitivity, F1-score, specificity, and AUC for XGBoost with five feature sets.

**Supplementary Table 6:** Table of LightGBM method performance with each feature set. The table contains the precision, recall/sensitivity, F1-score, specificity, and AUC for LightGBM with five feature sets.

**Supplementary Table 7:** miRNA-mRNA-lncRNA interaction network table of top feature miRNAs selected by feature importance analysis for across 14 cancer types.

**Supplementary Table 8:** List of miRNAs common with manually curated miRNA compendium. List of miRNAs common with miRNA isoform, miRNAs in EV, miRNAs in clinical trial and the exosomal miRNAs compiled from the literature.

**Supplementary Table 9:** Table of miRNA in ongoing clinical trial studies.

**Supplementary Table 10:** Table of miRNA-drug associations downloaded from noncoRNA DB for overlapping interacting miRNAs.

**Supplementary Table 11:** Table of GO enrichment analysis for all targets of 597 interacting miRNAs.

**Supplementary Table 12:** Table of KEGG pathway enrichment analysis for all targets of 597 interacting miRNAs.

**Supplementary Table 13:** Table of KEGG pathway enrichment analysis for all targets of 150 miRNAs from the RFE feature set.

**Supplementary Table 14:** Table of KEGG pathway enrichment analysis for all targets of 63 common miRNAs between miRNA compendium, CMC miRNAs, EV miRNAs, and 597 interacting miRNAs.

**Supplementary File 1:** Collection of miRNAs in cancer, clinical trial from literature. This collection is called miRNA compendium in this study.
